# Risk assessment reasoning and decision-making by ambulance professionals in patients with a transient loss of consciousness: a qualitative study

**DOI:** 10.1186/s13049-026-01598-1

**Published:** 2026-04-09

**Authors:** Lucia G. uit het Broek-Creemers, Lilian C. M. Vloet, Hester Vermeulen, Sivera A. A. Berben

**Affiliations:** 1 Research Department of Emergency and Critical Care, School of Health Studies, HAN University of Applied Sciences, Nijmegen, The Netherlands; 2 Scientific Institute for Quality of Healthcare, Radboud Institute for Health Sciences, Radboud University Medical Center, Nijmegen, The Netherlands; 3 Department of Primary and Community Care, Radboud Institute for Health Sciences, Radboud University Medical Center , Nijmegen, The Netherlands

**Keywords:** Emergency medical services, Transient loss of consciousness, Risk stratification, Decision-making, Clinical reasoning

## Abstract

**Background:**

Patients with a transient loss of consciousness (T-LOC) present a complex clinical situation for ambulance professionals. The challenge arises from the complexity of the diverse aetiologies associated with T-LOC and the demanding nature of the pre-hospital work environment. Insights into the experiences, dilemmas, and challenges faced by ambulance professionals in the risk assessment and decision-making in this patient category could help to understand the support they need. Our study aimed to explore the risk assessment reasoning and decision-making process of ambulance professionals in patients with a T-LOC in current practice.

**Methods:**

We conducted a qualitative study with focus group interviews in an emergency medical services (EMS) organisation in the Netherlands. Through purposive sampling, ambulance professionals were recruited for two online focus group interviews. An interview guide, based on literature and preliminary individual interviews, was used. The focus group interviews were transcribed verbatim and analysed using thematic analysis.

**Results:**

A total of eleven participants participated in two focus group interviews. Three main themes emerged: (1) *Clinical reasoning* covering the (initial) approach of ambulance professionals on scene, the T-LOC specific assessment, complexity of the aetiology of T-LOC, and the medical conveyance decision, (2) *Collaboration* describing the difficulties and facilitating factors in the interprofessional collaboration with other healthcare professionals in the chain of emergency care (e.g., general practitioners, emergency physicians, etc.) and the involvement of patients and relatives, (3) *Professionalism* describing the influence of professionals’ experiences (e.g. professional maturity), the sense of responsibility and accountability of the ambulance professional, and their subsequent need for reflection.

**Conclusion:**

Ambulance professionals follow a structured approach for the clinical assessment of a patient with a T-LOC. In addition to their professional experience, the complexity of the symptom and the patient’s context play a crucial role in the risk assessment reasoning and decision-making process. Ambulance professionals collaborate with other healthcare professionals to enhance their process, with varying experiences. There is a delicate balance between professionals’ sense of responsibility and accountability. A non-conveyance decision is considered precarious by professionals, and adequate support from management, education and interprofessional collaboration is required to feel confident in their decision-making.

**Supplementary Information:**

The online version contains supplementary material available at 10.1186/s13049-026-01598-1.

## Introduction

Patients with a transient loss of consciousness (T-LOC) pose a particular challenge for ambulance professionals and constitute up to 10% of emergency calls, with non-conveyance rates from 4 to 16% [1–4]. The challenge in patients with a T-LOC arises from its broad aetiology, which ranges from benign causes such as vasovagal syncope and psychogenic non-epileptic seizures to severe, potentially life-threatening conditions, such as fatal heart disease and subarachnoid haemorrhage [5, 6]. Professionals must differentiate between possible aetiologies and determine a patient’s risk for adverse events by identifying relevant signs and symptoms [5–7]. Signs and symptoms that may no longer be apparent when the ambulance professional arrives on the scene [7]. This complicates the risk assessment of this patient category, knowing there may be a life-threatening underlying condition [5, 6].

Additionally, the comprehensive nature of the pre-hospital work environment poses substantial challenges. Professionals in the pre-hospital work environment are often confronted with a tight timeframe, limited available information, and restricted resources which are inherent to the nature of the work [8–12]. These challenges are especially evident in the care for T-LOC patients due to the broad aetiology and associated risk assessment. In addition, professionals have to deal with interruptions and distractions arising from the patient’s situation and environment [8, 9]. These factors, which professionals may encounter to different degrees, can influence the decision-making process.

In relation to patient safety, the risk aversion of professionals also plays a role in the decision-making process. An ambulance professional’s response to risk is linked to their experienced support of management. When professionals do not feel supported by management, they will decide to mitigate their own risk of liability [10, 13–15]. Thus, they may sooner choose to transfer the patient to the Emergency Department (ED) due to the potential risk, even when it is possible to treat and release the patient on site. Conveyance of the patient to the ED is considered the safest option, as the responsibility of ambulance professionals in this scenario is transferred to the professionals in the ED [10, 11, 16, 17].

The decision-making process in pre-hospital emergency care is characterised by the need for professionals to maintain control in unpredictable conditions [18]. One way to maintain this control is the clinical reasoning process [8, 18]. To systematically describe this process, Dercksen et al. (2021) developed the “*SPART*” model. This model describes the entire reasoning process in different phases, consisting of S – start and situation at arrival, P – prologue and presentation of presenting complaint or symptom, A – anamnesis and assessment (including the ABCDE approach), R – reasoning and resolution, and T – treatment and transfer [8, 19]. These phases are presented in chronological order, recognising that in clinical practice, phases are often repeated, omitted, or performed in a different order [8]. This model can support the ambulance professional in the general decision-making process.

However, effective risk assessment and clinical decision-making in patients with a T-LOC in the pre-hospital setting may necessitate targeted support incorporating specialised medical knowledge. For the ED setting multiple decision rules for risk stratification and consecutive decision-making for this patient category have been developed; however for the pre-hospital setting, this kind of support is currently lacking [20]. Additionally, insight into the experiences, dilemmas, and challenges of ambulance professionals in risk stratification and decision-making in patients with a T-LOC was missing. Insights into these challenges could contribute to understanding the needs of ambulance professionals in risk assessment and decision-making for this patient category, as well as the development of appropriate support. Therefore, the study aimed to explore the risk assessment reasoning and decision-making process of ambulance professionals in patients with a T-LOC in current practice.

## Method

### Study design

We conducted a qualitative study with focus group interviews. We chose to conduct focus group interviews to gain a deeper understanding of the phenomenon of decision-making in patients with a T-LOC, allowing us to discuss the shared perceptions and experiences of ambulance professionals [21]. We followed the Consolidated Criteria for Reporting Qualitative Research (COREQ) to report our study (see Additional file 1) [22].

### Setting

The study took place in the Netherlands in an emergency medical services (EMS) organisation in October 2020. At the time of the research, this EMS organisation had 32 ambulances, divided over seven posts, which were deployed 40,039 times in 2020. The EMS organisation comprised 95 professionals with diverse backgrounds, consisting of 90 professionals with a Bachelor of Nursing and five professionals with a Bachelor of Health [23]. All professionals had obtained a qualification as specialised ambulance professionals. Specific details regarding the training of ambulance professionals and the staffing of ambulances in the Netherlands are described in a previous study [24].

In the Netherlands, ambulance professionals work according to the ‘National Protocol Ambulance Care (LPA)’. The LPA includes a protocol focused on T-LOC (syncope) (see Additional file 2). A medical supervisor is available 24 h a day, 7 days a week, for telephone consultation. Additionally, they can contact the hospital directly to consult with the ED physician, other medical residents, or specialists. They can also contact the patient’s general practitioner [25]. These consultations aim to gather additional information on patients’ medical histories for risk assessment or decision-making purposes, or to facilitate the transfer of care to a hospital or primary care setting. Based on clinical judgement, the LPA, and possible consultation, the ambulance professional provides the pre-hospital preliminary diagnosis and treatment and decides whether the patient should be conveyed to the hospital or not.

### Participant selection

We used purposive sampling to recruit participants. This approach allowed us to introduce a variety of experiences and perspectives of ambulance professionals on the risk assessment and decision-making in patients with a T-LOC, and facilitated an in-depth understanding of the topic [26, 27]. Participants for the focus group interviews were approached by a contact person (medical supervisor) within the EMS organisation. The medical supervisors were informed about the research’s aim. To reach a variety of experiences and perspectives, the medical supervisors were requested to inform and invite participants with diverse backgrounds (e.g., bachelor’s to master’s education and work experience in EMS) to participate in the study. Additionally, an information leaflet was distributed. We only received the e-mail addresses of participants who indicated they wanted to participate in the study. Therefore, we have no indication of the number of professionals who refused to participate. Participants who expressed interest in participating in the study were invited to attend the focus group interview. They were assigned to each focus group interview based on gender, as this was the only variable known to the research team beforehand. Participants were compensated for participating in the focus group interview during their leisure time with a small financial reward (i.e., €50.00).

### Procedures

Participants were invited to an online focus group interview, conducted via Microsoft Teams, lasting 90 min. An online focus group interview was chosen due to the COVID-19 regulations in place at the time. During the focus group interview, participants were asked to mute their microphones to reduce background noise and avoid distraction. However, participants were free to turn their microphones back on if they wished to speak, thereby enabling discussion to continue as much as possible. Two focus group interviews took place simultaneously, meaning they were run in parallel by different moderators. Two focus groups were selected to ensure that all participants remained visible on a single screen during the interviews, thereby facilitating optimal discussion and engagement. There were two moderators present per focus group interview. One moderator (LB and SB, both female) led the focus group interview, and the other (RE and BB, both male) took field notes and supported the first moderator. The moderators leading the focus group interviews were part of the research team and were therefore most familiar with the research subject and the EMS setting. This familiarity put them in the best position to ask substantive questions. All moderators had previous experience with qualitative research. At the time of the study, SB and RE were associate professors; they had a PhD and were Registered Nurses RN, BB was a lecturer-researcher, and LB was a RN and PhD student. SB and RE worked with the EMS organisation on other research projects but had no previous relationship with the individual ambulance professionals participating in this study. There was no hierarchical relationship between the participants. Participants worked for the same organisation. They sometimes knew each other personally, depending on whether they worked at the same ambulance station. Prior to the start of the focus group interview, the participants and researchers briefly introduced themselves to one another.

**Fig. 1:**
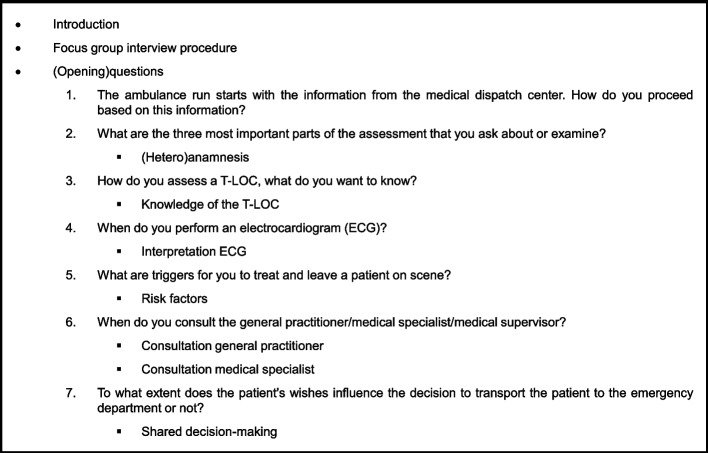
Interview guide

For the focus group interviews, an interview guide was developed in collaboration with the research team. This interview guide was based on literature, aetiology, and risk stratification in patients with a T-LOC, as well as clinical decision-making in ambulance care. In addition, preliminary individual interviews were conducted by bachelor student researchers, supervised by the same research team, to explore components of risk stratification and decision-making in patients with a T-LOC in pre-hospital practice. These preliminary individual interviews yielded six components that influence risk assessment and decision-making in patients with a T-LOC in pre-hospital practice. These components were actions arising from the medical dispatch centre notification, assessment of the patient, risk factors, decision-making, consulting with the medical specialist or general practitioners, and informing the patient and their relatives. The deeper meaning behind these components needed further exploration. Therefore, we used these components alongside the information from the literature review and the research team’s experiences to develop the interview guide for the focus group interviews (Fig. 1). The focus group interviews were videorecorded.

Participants received written information on the study’s purpose, privacy aspects, and the required time investment, and provided verbal consent prior to starting the video recording. In the invitation for the online focus group interview, participants were reminded that the session would be videorecorded. The focus group interviews began with a brief verbal summary of the written information by the first moderator, and verbal consent was obtained prior to start of the video recording.

### Data analysis

The focus group interviews were transcribed verbatim. Transcripts and findings were not returned to the participants for additional comments or feedback, in order to prevent (correction into) socially desirable responses. The focus group interviews were analysed following the steps of open, axial, and selective coding according to Boeije [27]. The analysis started with the open coding of the data. We used both inductive and deductive coding. A code tree was constructed prior to analysing the transcripts, based on literature and the components of the preliminary individual interviews, and meaningful units of the transcripts were assigned to those codes (deductive coding) (Additional file 3). Additionally, the researchers were free to add open codes to meaningful units that were relevant, but did not fit the original code tree (inductive coding). Units were considered meaningful when they related to the research question [27]. One researcher (LB) read the transcripts line by line and coded the first half of one transcript. Subsequently, the coding was reviewed in conjunction with a second researcher (SB), and the code tree was adjusted accordingly. The first transcript was coded again from the start by the first researcher and reviewed together with the second researcher. The process of open coding and reviewing was also applied to the second transcript. Differences in coding were discussed until consensus was reached.

The next step was the process of axial coding according to Boeije et al. [27]. The open codes and associated meaningful units were compared, and codes with similar meanings were grouped. If necessary, the original open codes were renamed. Subsequently, these new codes were aggregated, when related to the same subject. This resulted in more abstract and overarching codes, which formed the basis of the initial categories [27]. This step was performed by two researchers (LB and SB).

**Fig. 2:**
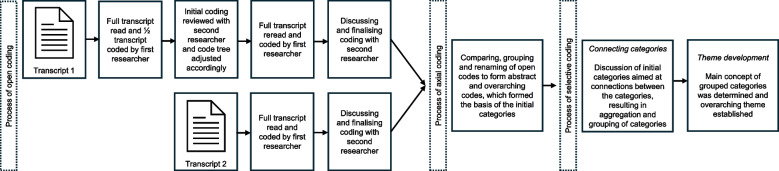
Visual presentation of the analysis process

The last step involved the process of selective coding, in which the initial categories were discussed in the research team along with the moderators. Field notes were incorporated into this step. The discussion was aimed at looking for connections between the categories in order to understand the deeper meaning and perspective in the data [27]. The research team and moderators discussed the meaning and different perspectives behind the categories and how they related to one another. This resulted in the merging and grouping of categories. Subsequently, the main concept of a group of categories was determined [27]. From this main concept, the overarching theme was established and the underlying categories served as sub-themes. The focus was on coherence rather than on the frequency of codes or categories. Analysis was performed using ATLAS.ti, version 9. The analysis process is illustrated in Fig. 2.

### Ethics

This study was approved by the Research Ethics Committee of the HAN University of Applied Sciences, ECO 481.07/23, as part of a larger research project. Ambulance professionals participated in the study voluntarily. Participants received information on the study’s purpose, privacy aspects, and the required time investment, and provided verbal consent prior to starting the video recording. Due to the COVID-19 regulations, which required us to conduct online focus group interviews, we opted to collect verbal consent instead of written consent.

## Results

Two focus group interviews were conducted with a total of 11 participants (five participants vs. six participants). One participant had a background in a Bachelor of Health, while the other participants had a background in a Bachelor of Nursing. Participants had a work experience ranging from 2 to 27 years, with a median of 11 years. Six participants were female. The duration of the focus group interviews was 83 min and 93 min.

Three main themes, ‘Clinical reasoning’, ‘Collaboration’, and ‘Professionalism’, were derived from the data, elaborating on the experiences of ambulance professionals with risk assessment and decision-making in patients with a T-LOC. These main themes are supported by nine subthemes (Table 1).

**Table 1:** Themes and subthemes

Theme	Subtheme
Clinical reasoning	Approach for (initial) risk assessment The T-LOC specific assessment Complexity of the aetiology of T-LOC Medical conveyance decision
Collaboration	Interprofessional collaboration Involvement of patients and relatives
Professionalism	(Professional) maturity Responsibility and accountability Reflection

### Clinical reasoning

Professionals perceive patients with a T-LOC as complex cases. They use various methods to manage patients with a T-LOC. This process involves distinct stages, including the collection of T-LOC-specific information, the approach and challenges of the risk assessment and conveyance decision-making process.

#### Approach for (initial) risk assessment

Drawing from the initial report, the professional forms an initial understanding of the patient’s situation. In cases of T-LOC, this assessment mainly relies on the patient’s age and surrounding context. Upon arrival, the professional makes a quick decision in the first seconds, based on first observations, whether immediate action is needed. If the patient appears critical, the professional uses the ABCDE method to stabilise the patient or transfer him as quickly as possible.

*“Mainly it’s the color and whether someone looks at you when you come in. If that’s not the case, then the alarm bells go off; yeah, along with a pale color (...) like this is, this looks bad, because then you, you have to really start following your systematic assessment. But if someone comes to you and talks to you, then you can park the ABCD methodology in the background for a bit and then you just go and have a conversation first.” (Respondent 5, Focus Group 1)*

If the patient is not in a critical condition, professionals will supplement their initial assessment, which was confirmed or revised upon arrival. They gather additional information on the T-LOC, explore differential diagnoses and, again, assess the patient’s age and surrounding context for potential risks.

*“You really try to include and consider everything as much as possible, and yes, you combine it with all the knowledge that you have and definitely you try to form as logical a story as possible for yourself and for the patient.” (Respondent 3, Focus Group 1)*

At all times, the professional keeps an open mind, to stay alert to sudden changes or unexpected developments. The professional evaluates both the somatic and psychosocial aspects of the situation to provide comprehensive care.

*“You just have to go in there with an open mind, and it can go in different directions. So I always go and see, that's kind of what I do by default.” (Respondent 4, Focus Group 2)*

#### The T-LOC specific assessment

For the risk assessment reasoning, professionals want to know precisely what occurred during and before the T-LOC. The professional needs to determine the extent and duration of the patient’s loss of consciousness. For this, the professional is mainly dependent on information from the patient or relatives, which can be complicated. The patient and relatives may not accurately assess the situation due to being more or less awake and alert, and their lack of training.

*“Unresponsive to the caller or a layperson is quite different than unresponsive to us. Look at it this way, if someone has out of it for a bit, that is very quickly described as unresponsive. While actually that patient is just fine, well maybe not quite in the best of spirits, but at least to us, still responsive enough.” (Respondent 2, Focus Group 1)*

Additionally, professionals indicate that a T-LOC is a dramatic event, often causing fear and panic among relatives, which can influence the responses of both the patient and relatives. To obtain adequate information on the loss of consciousness, the professional first reassures both the patient and their relatives. Only then can the professional continue an adequate assessment.

*“I always experience my work as half people management. And that’s just the way it is, especially with this patient category; it is a very important part of our work.” (Respondent 3, Focus Group 1)*

Next to the duration of unconsciousness, the presence of symptoms beforehand is a crucial factor. If the duration aligns with syncope, the focus shifts to assessing the risks associated with syncope.

*“It matters whether the patient felt it coming on. Or if it was out of the blue when he was suddenly on the floor, and that’s what you are trying to find out then, then. Or…or did something happen, or did he eat something, drink something? What, what caused him to be on the ground in that place? (Respondent 2, Focus Group 1)*

#### Complexity of the aetiology of T-LOC

Patients with a T-LOC are generally viewed as complex cases by professionals due to the wide range of potential causes. T-LOC is considered a broad symptom, and the challenge is to differentiate between the potential underlying risk factors. The variability in the signs and symptoms presented further compounds this complexity. As a result, professionals conduct a thorough examination before making a decision.

*“Only the problem is there could be 10 more reasons that those people are unresponsive for a while. And distinguishing between those, that's the crux I think (...) only some things still require a systematic approach so you can clearly distinguish and support [your choices].” (Respondent 4, Focus Group 1)*

The LPA T-LOC (syncope) protocol is a tool for ensuring all risk factors are considered. However, one professional found the protocol overly cautious, noting that patients are often not fully conscious or symptom-free. The protocol requires an ECG, but professionals may decide not to perform it under specific circumstances.

*“That depends a bit on the situation too. But that's what Respondent 1 also says, right? To what extent did you really faint, right? And if we check the pupils, and you squeeze your eyes shut, yeah, then it's a different story of course. But if that is not the case, then yeah, that [making an ECG] also applies.” (Respondent 5, Focus Group 1)*

#### Medical conveyance decision

The decision to treat and release the patient on scene depends on various factors. Professionals assess whether vital signs have normalised for that patient, and a thorough examination revealed no abnormalities. A clear, non-life-threatening explanation for the T-LOC also supports the decision not to convey. One professional indicated evaluating whether a hospital transfer would enhance the treatment at that time. Professionals considered non-conveyance runs as the most extensive and challenging runs due to the need for care evaluation and arranging of aftercare.

*“What also matters is will the hospital add something at that point? Well, often when I’m assessing [the situation] I don’t think urgent care will be given [in the hospital].” (Respondent 5, Focus Group 2)*

The decision to present a patient to the ED is twofold. First, the professional identifies sufficient medical reasons, such as persistent abnormal vital signs, the absence of symptoms prior to the T-LOC, or unexplained ECG abnormalities. Secondly, the professional’s worry plays a role. If doubts remain despite a thorough examination, the patient is transferred based on this worry, even without clear medical indications.

*“If I'm really unsure and I can't figure it out, then I send [the patient] in but I think, yeah no, I don't see a problem in that, no. And I always say it to the hospital then as well, I, I, I don't know, I can’t figure it out. I can't put my finger on it. I have a gut feeling, that's why.” (Respondent 2, Focus Group 2)*

Additionally, the complexity of the context plays a role in the decision-making process. Sometimes, the T-LOC is the reason for calling for assistance, but other factors may also contribute to the request for help at that moment. Furthermore, any substance use (drugs) as an aetiology of T-LOC can complicate the decision-making and the professional’s interventions.

*“There is a lot going on beforehand, you know? A situation, a home situation that actually isn’t going well, and this is the last straw. You know, it's never just the situation in itself.” (Respondent 4, Focus Group 2)*

### Collaboration

During the care for patients with a T-LOC, the professional interacts with the patient’s environment. She has regular contact with both the patient and relatives and other healthcare professionals related to the patient. These contacts range from informing, consulting and transferring.

#### Interprofessional collaboration

Professionals regularly consult with the general practitioner (GP). Contact with the GP is aimed at gathering information that the patient cannot provide sufficiently, such as medication use or prior episodes of a T-LOC, or transferring information and arranging aftercare when the professional decides to leave the patient on scene.

*“I think with this group, talking with the home, general practitioner, and especially [the patient’s] own GP really adds a lot of value because you only hear one side of the story at that moment. And GPs know the people, know their medical history. That's where you very often get information that justifies the decision or totally the opposite.” (Respondent 4, Focus Group 1)*

The experience of this contact depends on the GP. The professionals indicate it is sometimes difficult to reach an agreement, specifically when wanting to leave the patient on scene. The professionals transfer information to the GP and want to make follow-up appointments that the GP disagrees with. This requires the professional to exert extra effort in adjusting her plan or discussing with the GP whether to transfer the patient.

*“Sometimes it happens that we do want to send someone in and the doctor says I don't necessarily need to see him. Sometimes you want a kind of safety net, if it happens again within 12 hours or today, can the doctor take it over. And [the doctors] don't want that either.” (Respondent 1, Focus Group 2)*

The professional consults with the emergency physician if they expect that the emergency physician understands the problem better than the GP, such as in cases involving drug use. Contact is also generated when the professional wants to present the patient in the ED. This can create a new problem as she has to indicate for which specialism the patient is referred, which is not always clear in the case of a T-LOC.

*“There are also things that I absolutely don't discuss with a GP because I'm sure it will lead to sending [the person] in. And that's for example with drugs. This is better to consult with the ED doctor. He understands exactly what you are talking about (...) So the choice of specialist also determines to some extent whether or not someone is sent in.” (Respondent 5, Focus Group 2)*

### Involvement of patients and relatives

The professional involves the patient and relatives at various levels. First, she explains the decision-making process and involves the patient and their relative in the process, as it were. Then, she can consult with them to verify whether they agree with the decision-making process. Lastly, she can actively involve the patient and their relatives, incorporating their wishes into the decision-making process.

*“The patient in any case has to have a good feeling when we leave, with what the decision is. And also, it has to be very clear to them so they're not going to be calling in again a half hour later. Because, of course, that's also what can happen.” (Respondent 4, Focus Group 2)*

When deciding to release the patient on scene, the professional will discuss with the relatives the possibilities of observing the patient for a period. If this is not possible, she may choose to transfer the patient to the ED because aftercare is insufficiently guaranteed.

*“If you really have a, a doubt then I could still discuss with the family how they see it, and whether there is a daughter or someone else in the house all day. Well, then leaving someone like that is different than someone staying home alone.” (Respondent 5, Focus Group 2)*

### Professionalism

The professionalism of the ambulance professional plays a role and can influence clinical reasoning, risk assessment, and decision-making. Professionalism can be distinguished in three influencing factors. Starting with the (work) experience of the professional, followed by the balance between sense of responsibility and accountability, which leads to the need for reflection.

#### (Professional) maturity

In the patient’s assessment, the professional’s (gut)feeling and clinical judgement are important factors. The professional’s assessment in that first second upon arrival is made based on their clinical judgement.

*“And you do have to be certain, especially with yourself, that you made the right choice. And yes, like I said, I think if you don't have that much experience, that, that is just very difficult…when you're still starting out.” (Respondent 3, Focus Group 1)*

The novice professional is still developing her clinical judgement and is therefore more dependent on tangible data. This makes clinical reasoning difficult for patients with a T-LOC because tangible data are often missing when the professional arrives. As a result, the novice professional adheres more strictly to the protocol.

*“I still work very protocolled. And I certainly would leave people at home if there's a clear indication for it, but if I don't know and can't get the complete picture, then somebody is quickly transferred. Yeah.” (Respondent 5, Focus Group 1)*

#### Responsibility and accountability

The decision to leave a patient on scene, despite any consultation with other professionals, remains the responsibility of the professional. For the patient with a T-LOC, this means they examine the patient extensively, so they are also accountable to themselves for the decision taken.

*“And obviously if there is a collapse, then of course in that moment you thinking a certain way. But you really have to ask everything. You want to make sure that you could leave somebody at home.” (Respondent 4, Focus Group 2)*

However, the professionals also indicate they cannot give a 100% guarantee that nothing will happen if the patient is left on the scene. A certain risk is always taken. This risk is inherently linked to their work and is the added value of their profession. They want to be able to make the decision to leave the patient on scene despite the possible risk taken.

*“That's a very important point for me, though. I have, I'm not God, right? I can't, I can't guarantee people that, no, nothing happens. Sometimes people are like, am I going to die? Then I think, yes, I don't know (...) And by now I am in such a position that I think, yeah, I can't give a 100% guarantee. Nobody can.” (Respondent 2, Focus Group 2)*

This personal responsibility, combined with the potential risks, means the professional can be held accountable for their actions by both the patient, relatives, and management. This possibility can sometimes cause them to adjust their actions and decision-making. Occasionally, professionals can dwell on their choice when leaving a patient on scene and ‘hope’ they will not be held accountable for it later.

*“I hope you guys have had that too. That sometimes you left people at home, and you just cross your fingers. And that you think, um I'm not quite sure, and I hope that I, that I never hear about this again.” (Respondent 2, Focus Group 2)*

#### Reflection

Professionals reported a need to reflect and discuss cases in which they have been involved. However, they indicated that they currently have insufficient opportunities to reflect on and discuss cases in pre-hospital ambulance practice. The professional has made her decision based on the information at hand and does not receive any follow-up information on the case, as the treatment relationship between the professional and patient has ended. Moreover, the professional does not receive any feedback on whether her choice and actions were based on the correct assumptions and knowledge. This is particularly complicated in T-LOC patients due to the different causes.

*“Well what [the other respondent] says about there being a need, certainly. That sometimes you actually just want to know what came out of it. I do too. So yes, definitely over the years, yes, you accept that, that you just don't find out.” (Respondent 4, Focus Group 2)*

*“I think it is very important that you share such things with each other, right? You, you learn from each other, the choices that one another make. That also applies to non-conveyance. What did I forget and what didn’t I forget? What did I do well and what can someone else learn from it? In our work, we almost never talk about that. We don't talk about that. Yeah. Because is it still, yeah, the sword of Damocles hanging over you.” (Respondent 3, Focus Group 2)*

## Discussion

This study aimed to explore the experiences of ambulance professionals regarding risk assessment reasoning and decision-making in patients with a T-LOC in current practice. The findings show that professionals follow a certain methodology for the risk assessment, with a focused attention on information relevant to T-LOC. They view T-LOC as a complex symptom due to its varied underlying causes and dependency on the patient and relatives for specific information. However, the decision-making process is not solely based on high- and low-risk patient factors; it is influenced by contextual factors, such as the collaboration with other healthcare professionals and the ability to arrange aftercare for the patient. Moreover, the professionals feel a strong sense of responsibility, but this also means they can be held accountable for their decisions. This interplay creates a need for self-reflection among the professionals.

The approach to risk assessment of patients with a T-LOC, as described by the professionals, is consistent with the phases and activities of the *SPART* model [8]. Although not surprising given the same context, in the Netherlands, it is clear that professionals desire a structured assessment for an elusive symptom such as a T-LOC, when all tangible symptoms have already passed. Professionals want to examine a patient with a T-LOC systematically and thoroughly before making a conveyance decision, especially when considering leaving the patient on scene to minimise the patient’s risk. Current training and protocols in ambulance care are more focused on identifying patients in critical condition, rather than supporting the decision to leave a patient on scene. Recent studies have shown that there is a need for the development of clear treat and release criteria or protocols, as well as education on non-conveyance assessment and decision-making, to enhance patient safety and increase the comfort of ambulance professionals [15, 28]. At the same time, the actual development of training and protocols remains limited [29].

In the decision-making process, professionals often collaborate with GPs, which can lead to varying experiences, especially when considering leaving a patient on scene. A possible explanation for these varied experiences could be the unfamiliarity of GPs with the scope and practice of ambulance professionals, as well as the perceived lack of experience and knowledge of these professionals by GPs [30, 31]. This challenge is particularly pronounced in patients with a T-LOC, given the diverse aetiology of such cases [5]. Moreover, the GP’s absence on scene can create additional difficulties, as they are entirely dependent on the ambulance professional’s assessment. Different perspectives on the responsibility of patient care and the potential transfer of that responsibility may further complicate that collaboration [17, 30, 31]. In pre-hospital emergency care for older patients with complex needs, key factors influencing interprofessional collaboration were identified as a shared understanding of patient care objectives, the necessity of timely and accurate patient data, and the need for well-defined roles [32]. To address these challenges, interprofessional training could be a solution to improve collaboration between GPs and professionals. Such training could enable GPs to understand the competencies of ambulance professionals, while enhancing shared understanding, clarity in roles and responsibilities, and strengthening teamwork [32–35]. Additionally, interprofessional training could lead to enhanced communication [36].

The findings indicate a delicate balance between professionals’ sense of responsibility and accountability and their need for adequate support. While professionals are willing to take responsibility for their decisions, knowing they can be held accountable for the outcome, this awareness can still influence their decision-making process. Professionals may face an ethical dilemma when trying to make the ‘right’ choice. This interplay between risk-taking and risk aversion appears to be a significant factor affecting decision-making in pre-hospital care, regardless of the patient category [10, 11, 16]. When ambulance professionals adopt risk-averse decision-making strategies, this hinders the delivery of appropriate care and results in potential overuse (e.g. transporting patients even when sufficient treatment can be provided on scene) [37]. It requires bravery and nursing leadership of professionals to treat and release a patient on scene. In the context of today’s healthcare, where the interests of the patient and patient safety are paramount, it is essential that they are equipped to do so [38]. This highlights the need for professionals to receive adequate managerial support [13, 14, 18], which is essential for feeling confident in their decision-making and enabling opportunities for mutual learning and reflection. This need, identified in the results and supported by literature, is critical for the development of knowledge and skills [18].

### Strengths and limitations

One limitation of the study is that the focus group interviews were conducted simultaneously by different moderators. This means we did not have the opportunity to build on the results of an earlier focus group interview, and there could be no consultation between the moderators of the two focus group interviews. Additionally, all participants who indicated they were willing to participate were invited to one of the focus group interviews. The number of participants was fixed. Data analysis showed the themes emerged from both focus group interviews, but we could not perform an additional focus group interview to confirm thematic saturation, meaning that in the data analysis process repetitive codes and themes were identified, and no new information or relationships emerged [39]. However, in terms of information power, our study has a relatively specific aim and sample, and data collection and analyses were guided by literature, implying in this case a smaller sample size is justified [40]. Additionally, we used exploratory individual interviews to gain an initial idea of possible themes that could arise and collect preliminary data. Through the focus group interviews, performed by experienced moderators, we gained a deeper understanding of these themes and were able to discuss the preliminary findings, which ensured that we collected rich data. This suggests adequate information power has been reached in our study [40].

The focus group interviews were not analysed completely independently by two researchers. To prevent bias, a systematic approach was chosen in which open coding, axial coding, and initial categorisation were done by one researcher, while a second researcher acted as a reviewer. Additionally, the final themes were discussed with the researchers involved in the focus group interviews, in collaboration with the research team.

The moderators who conducted the focus group interviews had expertise in the subject and the pre-hospital context but no personal involvement with the professionals. Their understanding of the subject and context enabled them to ask thoughtful questions and highlight the underlying experiences. Since they did not personally know the professionals, they were able to approach the subject with openness.

The focus group interviews were conducted during the COVID-19 pandemic. We do not expect this to have affected their answers regarding their risk assessment reasoning and decision-making process in patients with a T-LOC. However, it could have influenced their experiences with interprofessional collaboration, as all healthcare professionals experienced a high level of workload, which could hinder effective collaboration. Additionally, because of the COVID-19 pandemic, the focus group interviews were held online. Although we were able to conduct our study, it did require some adjustments compared to face-to-face focus group interviews. Participant interaction, nonverbal cues, and collection of in-depth data could be hindered by the online format [41, 42]. In our study, we facilitated participant interaction by asking participants to turn on their camera and all the participants were visible on one screen during the meeting. Therefore, we had to adjust the number of participants per focus group interview to a maximum of six. The video recording made it possible to observe nonverbal expressions, and to respond to them as moderators. To enable discussion between participants, they were free to turn on their microphones if they wished to speak. This could result in participants speaking at the same time, which implied that the moderator in those situations had to intervene. Potentially, this could be disruptive for the discussion, on the other hand it more closely approximated the natural dynamics of a face-to-face focus group discussion. As a disadvantage, because participants participated from their own environment, they were sometimes more easily distracted by their home environment, which made the moderator’s summarisation more important. Fortunately, we did not experience any technical difficulties in the online focus group interviews. Overall, we experienced an adequate participant interaction. Participants spoke freely and actively responded to each other. Participants were recruited from a purposive sampling approach. The sampling approach resulted in the inclusion of participants with relevant expertise from diverse perspectives in terms of gender, years of work experience, and professional background. Participants were assigned to each focus group interview based on gender, as this was the only variable of participants known beforehand. Subsequently, there also appeared to be a sufficient distribution of the other variables in each focus group interview. This inclusion of participants with expertise from diverse perspectives and the distribution in the focus group interviews allowed us to collect rich and detailed data. However, this approach may lead to selection bias, as the sample may not be representative of the broader population. Therefore, our data should be placed within its specific context and valued for the insight it provides, not primarily for its generalizability. Additionally, participants were recruited by the medical supervisors who hold a superior position to the ambulance professionals. This could have influenced their willingness to participate. However, even if this had occurred, we did not notice it during the focus group interviews and do not expect it to have influenced their answers. Ambulance professionals were fully aware that their responses were used solely for research purposes and that there would be no feedback of individual responses to the medical supervisors.

## Conclusion

Ambulance professionals work methodically to assess the risk of a patient with a T-LOC thoroughly. This thoroughness is necessary because T-LOC is considered a complex symptom, and the assessment is further complicated for professionals, as they rely on information from the patient or relatives. Nevertheless, decision-making is not solely based on high- and low-risk patient factors, but is also influenced by contextual factors, such as collaboration with other healthcare professionals. Additionally, professionals have a strong sense of responsibility and balance their responsibility with subsequent accountability. Therefore, they need opportunities for reflection with their peers. A non-conveyance decision in patients with a T-LOC is considered precarious. This highlights the need for adequate managerial support, interprofessional collaboration, and education regarding non-conveyance decision-making for ambulance professionals.

## Supplementary Information

Additional file 1.

Additional file 2.

Additional file 3.

## Data Availability

The datasets generated and/or analysed during the current study are not publicly available due to the sensitivity and possible traceability of the participants due to the qualitative nature of the data but are available from the corresponding author on reasonable request.

## Abbreviations

ECG: Electrocardiogram
ED: Emergency department
EMS: Emergency medical services
GP: General practitioner
LPA: National Protocol Ambulance Care
T-LOC: Transient loss of consciousness
