# Supplementary material for: Risk assessment reasoning and decision-making by ambulance professionals in patients with a transient loss of consciousness: a qualitative study
Source: Scand J Trauma Resusc Emerg Med. 2026 Apr 9;34:97. doi: 10.1186/s13049-026-01598-1 (PMC13217765; doi:10.1186/s13049-026-01598-1)
Supplement: Supplementary file 3 — Additional file 3. [file 13049_2026_1598_MOESM3_ESM.pdf]

Additional file 3

Data-analysis: Code Tree

SPART model (1)

- Start & Situation
  - Start → interpretation of information provided by the dispatch centre
    - Code: preparation / notification medical dispatch centre
  - Situation → first subjective, and intuitive interpretation of the scene
    - Code: Initial image of patient
- Prologue & Presentation
  - Prologue → retrospective interpretation of factors leading to and influencing the presenting complaint
  - Presentation → indicating the reason for the call for assistance
    - Code: TLOC
- Anamnesis & Assessment
  - Anamnesis → medical history tacking
    - Code: (hetero)anamnesis
    - Code: consultation general practitioner or emergency physician
  - Assessment → general and specific physical examination
    - Code: Methods
      - ABCDE
      - Tract anamnesis
- Reasoning & Resolution
  - Reasoning → the process of ordering, evaluating, and interpreting of the information and detect information deficiencies
    - Code: complexity
    - Code: age & context
    - Code: risk factors
    - Code: responsibility for decision-making
    - Code: professional's sense of worry
    - Code: professional's experience

        - Code: protocol LPA T-LOC
    - Resolution
        - The (clinical) decision on what to do
            - Code: decision-making
                - Shared decision-making
                - Argumentation
- Treatment & Transfer
    - Treatment → therapy guided by protocols and guidelines of the LPA
    - Transfer → formal closing of the ambulance consult
        - Code: Information and handover
            - Aftercare
            - Creating network
            - Handover(form)

(1) Dercksen B, Struys MMRF, Cnossen F, Paans W. Qualitative development and content validation of the “SPART” model; a focused ethnography study of observable diagnostic and therapeutic activities in the emergency medical services care process. BMC Emergency Medicine. 2021;21(1)
